# Glycolipids implicated as mediators of clinically visible retinal pigment epithelial migration in age-related macular degeneration

**DOI:** 10.1073/pnas.2503191122

**Published:** 2025-07-14

**Authors:** Zhen Wang, David M. G. Anderson, Jeffrey D. Messinger, Christine A. Curcio, Kevin L. Schey

**Affiliations:** ^a^Department of Biochemistry and Mass Spectrometry Research Center, Vanderbilt University, Nashville, TN 37532; ^b^Department of Ophthalmology and Visual Sciences, University of Alabama at Birmingham, Birmingham, AL 35233

**Keywords:** age-related macular degeneration, retinal pigment epithelium, imaging mass spectrometry

## Abstract

The retinal pigment epithelium (RPE) is the metabolic gatekeeper to the photoreceptors, thus playing many essential roles in healthy vision. Under certain conditions, RPE cells may transdifferentiate and migrate from the RPE layer. Ectopic RPE cells are potential signal sources for hyperreflective foci, prominent clinically visible biomarkers in age-related macular degeneration, which causes central vision loss globally. Applying multiple imaging modalities including ex vivo optical coherence tomography, autofluorescence microscopy, and imaging mass spectrometry (IMS) to human retina tissue, we compared lipid profiles in ectopic and orthotopic RPE cells. Our results showed that ectopic RPE cells share some molecular signatures with normal RPE cells as revealed through autofluorescence imaging and IMS. In both orthotopic and ectopic RPE cells, IMS detected phosphatidylglycerol, PG 36:2, and several triacylglycerols containing long-chain fatty acids. GM3 gangliosides (40:1, 42:1, and 42:2) were also detected in ectopic RPE cells with differences in abundance in different populations of ectopic RPE cells. Lactosylceramide (LacCer 44:5) and glucosylceramide (GlcCer 44:5) were found exclusively in ectopic RPE cells. In contrast, ectopic RPE did not exhibit signals of phosphatidylinositols (PI) (PI32:0, PI32:1, PI34:1, and PI34:2) normally detected in RPE cells. Near-single-cell resolution IMS results suggest that RPE transdifferentiation, with loss of normal functions and gain of new functions like migration, may be linked to altered metabolism of glycosphingolipids and PIs.

## Significance

In age-related macular degeneration, clinically visible biomarkers (hyperreflective foci,HRF) may represent retinal pigment epithelium (RPE) cells that transdifferentiated and migrated into neurosensory retina. Under such conditions, functional properties may be lost or gained. By comparing lipid signals of orthotopic and ectopic RPE at near-single-cell resolution, we found that ectopic RPE retains some RPE-specific lipids such as very-long chain fatty acid containing triacylglycerides. However, altered glycosphingolipids and phosphatidylinositol (PI) metabolism may drive RPE cell migration. Ectopic RPE cells have elevated levels of lactosylceramide, glucosylceramide, and GM3 gangliosides, while showing lossof several PIs present in native RPE cells. These changes could be linked to inflammation, potentially triggering epithelial-to-mesenchymal transition, RPE cell migration, and appearance asHRF in optical coherence tomography.

Advances in optical coherence tomography (OCT), a noninvasive clinical imaging technique showing tissue cross-sections, have significantly enhanced our understanding of retinal disease. As detected by OCT, hyperreflective foci (HRF) are small, discrete, punctate lesions of high reflectivity in a single or clustered distribution (1, 2). HRF are observed under disease conditions such as diabetic macular edema (3), uveitis macular edema (4), retinitis pigmentosa (5), and retinal vein occlusion (6). HRF have been extensively studied by histology in age-related macular degeneration (AMD) (7–9). As defined by a clinical consensus group, HRF in AMD are at least three pixels wide in OCT (~23 µm equivalent diameter) and are as reflective as the native layer of retinal pigment epithelium (RPE) cells.

OCT allows individual lesions to be precisely tracked over time, illuminating both local and overall disease progression. As such, HRF may represent strong biomarkers for disease advancement and response to treatment (3, 10). The presence of HRF was one of four biomarkers proposed for a composite risk score to predict AMD progression (8). In longitudinal studies (11–13), HRF are the major predictor of progression and often associated with the apices of drusen (14), the pathognomonic extracellular lesion of AMD, appearing in all retinal layers, and increasing in number in later disease stages (15, 16).

Proposed correlates to HRF in AMD include lipid exudates (3), activated microglial cells (17, 18), degenerated photoreceptor cells (19), and migrating RPE cells (7, 8, 20). Intraretinal HRF colocalize with pigment clumping in color fundus photography (20–22). HRF often appear vertically above disturbances of the RPE layer atop drusen (7, 8) and move from outer to inner retinal layers during follow-up observation (7, 8). These data support the hypothesis that at least some HRF originate from RPE cells migrating into the neurosensory retina (7, 8, 20). This hypothesis does not exclude the possibility of non-RPE cells contributing to HRF, as shown in neovascular AMD (17).

RPE cells are postmitotic but can migrate and enlarge under adverse conditions (20, 23). Pigmented cells colocalized with HRF are believed to be transdifferentiating RPE. These cells lack immunoreactivity for characteristic retinoid marker RPE65 yet gain immunoreactivity for immune markers CD68 and CD163 (8, 24), a process that begins within the RPE layer (8). Molecular continuity between in-layer (orthotopic) and out-of-layer (ectopic) RPE is suggested by a continuity of lengthening fluorescence lifetimes, due to differences in fluorophores and/or the intracellular environment (25). Ultrastructural continuity is supported by a lack of phagolysosomes enclosing RPE organelles, to be expected if HRF represented phagocytes that engulfed RPE (26). Epithelial–to-mesenchymal transition (EMT) is proposed as a process that could cause RPE migration. Accordingly, characteristic EMT molecular markers (vimentin and transcription factor Snail) are detected in lysates of AMD eyes (27).

Comparing the molecular composition of ectopic to orthotopic RPE, in relation to surrounding areas, may help confirm RPE transdifferentiation and migration as well as provide insights into driving mechanisms. Alterations in lipid metabolism have been suggested as a key EMT mechanism in disease (28). In addition, the linkage between lipids and AMD is strong, given the accumulation of RPE-originated lipoprotein-derived lipids in high-risk drusen (26, 29, 30) and an association of AMD with variations in genes of the HDL pathway (31, 32). A detailed spatial lipidomic analysis can elucidate lipid signatures associated with RPE migration and the local lipid environment. Imaging mass spectrometry (IMS) is particularly suited for this purpose, as it has high sensitivity and can map the spatial distribution of hundreds of lipids at near-single-cell resolution (33, 34). Herein, we combined IMS with other imaging modalities, including fluorescence microscopy and ex vivo OCT, to investigate, at the molecular level, cells corresponding to HRF. The primary goal was to compare lipid signals between orthotopic and ectopic RPE to identify processes that may trigger transdifferentiation and migration. Our results implicate glycolipids in RPE migration and the presence of clinically visible HRF in AMD retina.

## Results

### Multimodal Imaging of HRF and Ectopic RPE Cells.

**Fig. 1.**
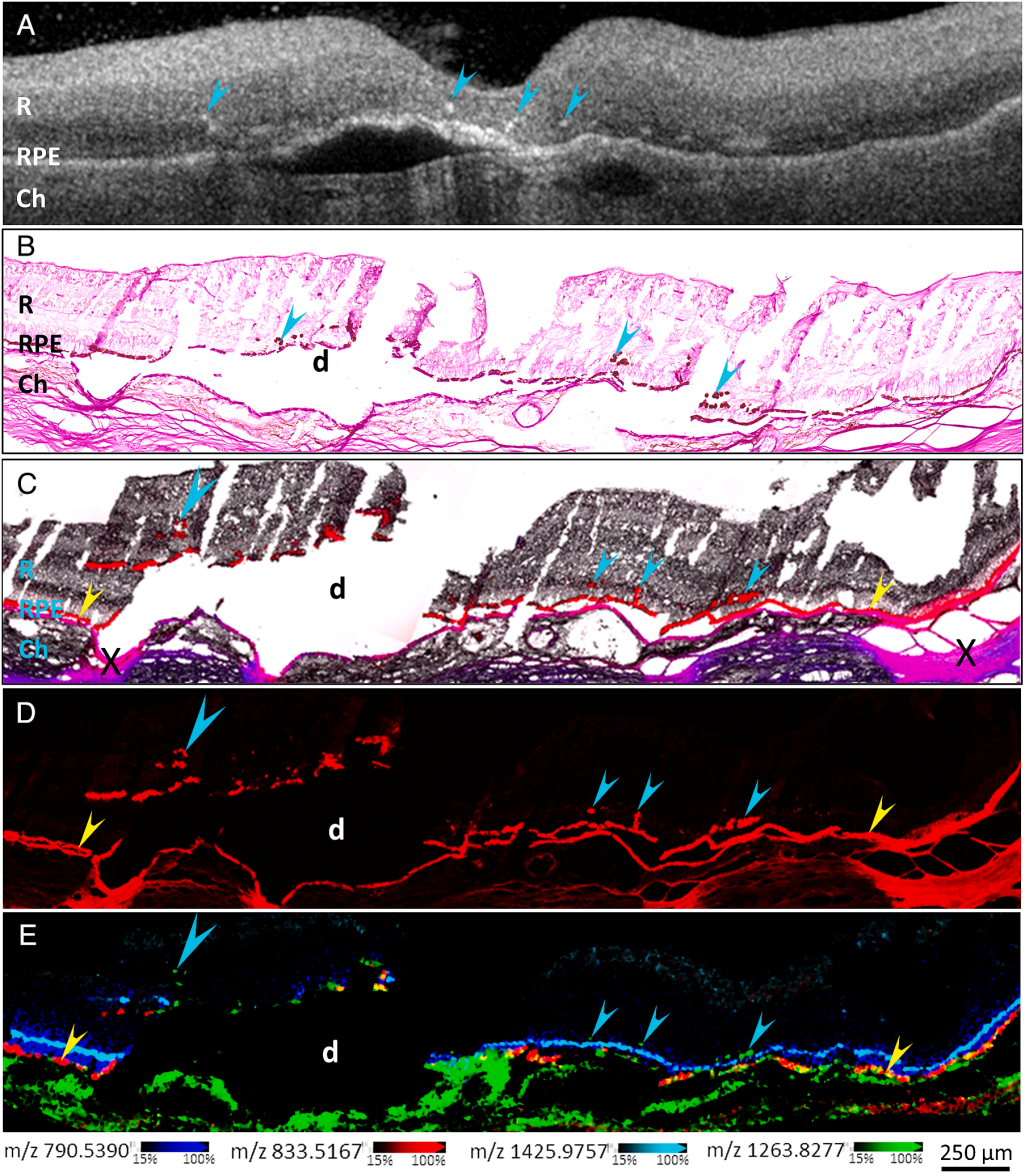
Multimodal imaging of HRF and ectopic RPE cells. The ex vivo OCT B-scan image from Donor 1 (*A*) indicates the presence of HRF in this donor eye with AMD (cyan arrowheads). Retina sections were stained with PASH and the transmission bright-field image is shown in (*B*). Neighboring sections were imaged in brightfield (*C*), scanned for autofluorescence (*C* and *D*), and analyzed by IMS (*E*). PASH stained sections showed the presence of drusen (d) whose contents were mostly dislodged and migrating RPE cells (cyan arrowheads). Merged bright-field and autofluorescence images (*C*) show the location of native RPE (red fluorescence, yellow arrowheads), Bruch’s membrane [purple, overlay of DsRed and DAPI channels, also showing tissue folds (X)] and ectopic RPE cells (cyan arrowheads). The DsRed channel of autofluorescence is shown in *D*. IMS images, acquired in negative ion mode, in *E* shows the overlay of four lipids that located in the photoreceptors OS and IS (PE 40:6, blue), IS (CL 70:5, cyan), orthotopic RPE cells (PI 34:2, red), or orthotopic and ectopic RPE cells (GM3 42:1, green). The signal shown in green is also present in choroid. Both *C* and *E* showed the ectopic RPE cells (large cyan arrowhead) migrated further inward, away from the photoreceptor IS. R: Retina, Ch: Choroid.

We imaged retina and choroid tissue from donors with AMD (Table 1) with multiple techniques. Eyes were selected based on the presence of HRF in ex vivo OCT B-scans. The diagnosis of AMD and presence of ectopic RPE cells was confirmed by brightfield examination of periodic acid–Schiff hematoxylin (PASH)-stained tissue sections, as well as autofluorescence imaging. As shown in Fig. 1 (Donor 1), an ex vivo OCT B-scan (Fig. 1*A*) and PASH stained tissue (Fig. 1*B*) illustrate the presence of HRF and pigmented cells in the neurosensory retina, respectively. PASH staining employed a section adjacent to that used for IMS and therefore represented similar but not identical ectopic cell distributions. However, brightfield microscopy, fluorescence microscopy, and IMS imaging (Fig. 1 *C*–*E*, respectively) on the same tissue section allowed accurate coregistration across multiple modalities. High spatial accuracy is crucial not only for assigning IMS signals to orthotopic versus ectopic RPE at different heights in the retina but also for determining what adjoining structures in tightly packed outer retina do not have those signals. Note that we use cyan arrowheads and yellow arrowheads to highlight ectopic and orthotopic RPE, respectively, in all figures. All lipid signals represented in IMS figures are summarized in Table 2.

**Table 1.** Human donor eyes

Donor	Age (y)	Systemic condition	AMD status	Figures and Tables
1^*^	97	n.a.	Atrophic AMD	Figs. 1–7 and *SI Appendix*, Figs. S2, S3, and S5, Table 2
2	93	Coronary artery disease, hypertension, rheumatoid arthritis, anemia	Early-intermediate AMD	Figs. 2, 3, 5, and 7 and *SI Appendix*, Figs. S2 and S5, Table 2
3	97	Atrial fibrillation, myocardial infarct, pacemaker, renal failure, osteoporosis	Early-intermediate AMD	Fig. 3 and *SI Appendix*, Fig. S5, Table 2
4	80	Acute respiratory failure, cardiomegaly, pulmonary vascular congestion, hypertension, depression, anxiety, mastectomy, hysterectomy	Early-intermediate AMD	Table 2
5	83	Dementia, Alzheimer’s, hypertension, congestive heart failure	Early-intermediate AMD	Table 2

All donors were white and female, except for Donor 5, who was a white male. All eyes are right eyes.

^*^Indicates DMACA matrix was used for both positive and negative mode IMS analysis; DAN was used for negative mode and DHA was used for positive mode for the other samples.

**Table 2.** Lipid identification and m/z values detected by IMS

		Experimental m/z ([M-H]^−^) (negative mode)					
		Donor 1		Donor 2		Donor 4	
Lipids	Theoretical m/z [M-H]^−^	m/z	ppm error	m/z	ppm error	m/z	ppm error
PG 36:2	773.5338	773.5341	0.38	773.5339	0.13	773.5320	2.33
PE 40:6	790.5392	790.5390	0.25	790.5399	0.89	790.5377	1.90
PI 32:1	807.5029	807.5011	2.23	807.5027	0.48	807.4991	4.71
PI 32:0	809.5186	809.5175	1.36	809.5159	3.33	809.5147	4.82
PI 34:2	833.5186	833.5167	2.28	833.5163	2.76	833.5164	2.64
PI 34:1	835.5342	835.5340	0.24	835.5332	1.20	835.5330	1.44
GM3 40:1	1,235.7999	1,235.7984	1.21	1,235.7992	0.57	1,235.7959	3.24
GM3 42:2	1,261.8154	1,261.8140	1.11	1,261.8148	0.48	1,261.8138	1.27
GM3 42:1	1,263.8311	1,263.8277	2.69	1,263.8287	1.90	1,263.8302	0.71
CL 70:5	1,425.9806	1,425.9757	3.44	1,425.9811	0.35	1,425.9747	4.14
Lipids	Theoretical m/z [M + H]^+^	Experimental m/z ([M + H]^+^) (positive mode)					
		Donor 2		Donor 3		Donor 5	
		m/z	ppm error	m/z	ppm error	**m/z**	**ppm error**
DG 38:6	641.5139	641.5120	2.96	641.5127	1.87	641.5150	1.7
GlcCer 44:5	832.6661	832.6642	2.28	832.6626	4.20	832.6624	4.4
LacCer 44:5	994.7189	994.7193	0.40	994.7147	4.22	994.7174	1.5
TG 68:4	1,079.9940	1,079.9996	5.19	1,079.9964	2.22	1,079.9982	3.9
TG 80:16	1,223.9940	1,224.0004	5.23	1,223.9955	1.22	1,223.9964	1.6
TG 84:20	1,271.9940	1,271.9963	1.81	1,271.9955	1.18	1,271.9965	2.0

As reported (34), lipids exhibit layer-specific distributions in the neurosensory retina that can guide characterization of the height of RPE migration. As shown in Fig. 1*E*, three lipid signals correspond to adjacent and distinct retina layers: PI 34:2 at m/z 833.5167 (red) in orthotopic RPE, PE 40:6 at m/z 790.5390 (blue) among photoreceptor inner (IS) and outer segments (OS) and predominantly in the OS, and cardiolipin CL 70:5 at m/z 1425.9757 (cyan) at the level of photoreceptor IS mitochondria. None of these three signals were detected in ectopic RPE cells. Reduced OS layer thickness can be clearly observed from signal of m/z 790.5390 (blue) above the atrophic area where RPE migration occurs. Based on Fig. 1*E*, ectopic RPE cells (small cyan arrowheads) were located slightly above the CL 70:5 signal layer, confirming a migration from their original layer. Others (large cyan arrowhead) are further away from CL70:5, indicating further migration into the neurosensory retina. IMS signals in ectopic RPE cells were identified by comparing mass spectra at specific pixels to the mass spectra corresponding to orthotopic RPE cells. For example, the ganglioside GM3 42:1 signal at m/z 1263.8277 (Fig. 1*E*, green) was found in ectopic RPE cells. This ganglioside was also detected in orthotopic RPE cells and choroidal cells.

### Ectopic and Orthotopic RPE Cells Share Lipid Signals.

**Fig. 2.**
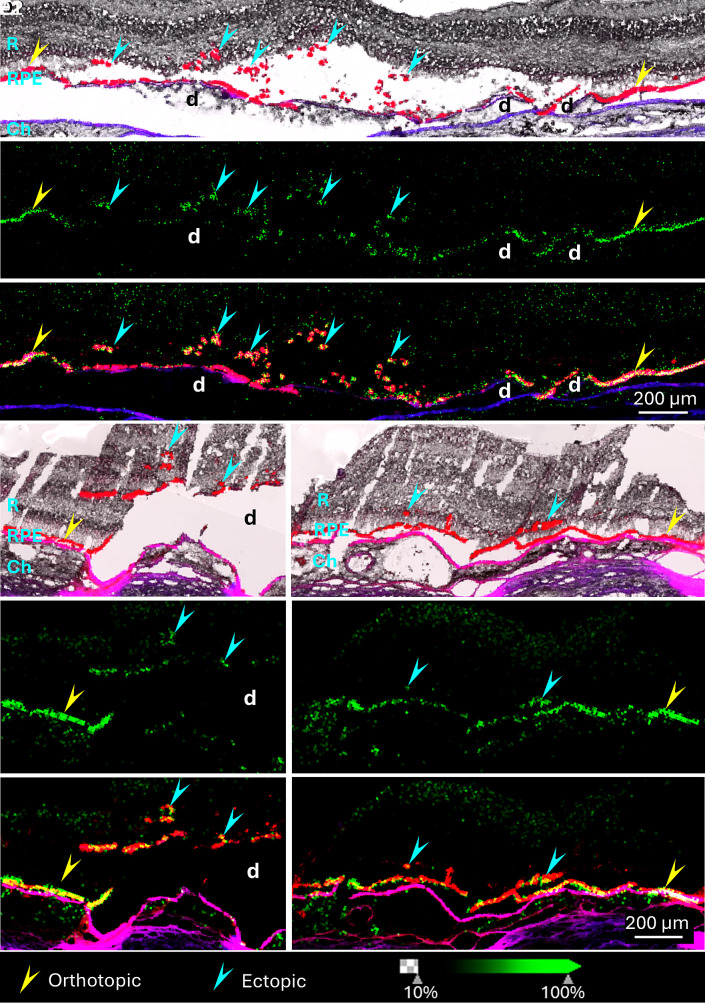
PG 36:2 is present in orthotopic and ectopic RPE cells in Donor 1 (*A*–*C*) and Donor 2 (*D1*–*F2*). Overlays of autofluorescence signal (red) and brightfield images (*A*, *D1*, and *D2*) in samples obtained from two donors (Donor 1 and 2) show the presence of orthotopic RPE cells (yellow arrowheads) and ectopic RPE cells (cyan arrowheads). Bruch’s membrane is shown in purple (overlay DsRed and DAPI channels). IMS signal (green) corresponding to m/z 773.5339 (*B*, *E1*, and *E2*) is detected in RPE from Donor 2 (*A*–*C*) and Donor 1 (*D1*–*F2*) using negative ion mode. An overlay of the IMS signal and DsRed autofluorescence signal (Donor 2 in panel *C* and Donor 1 in panels *F1* and *F2*) shows the colocalization of PG 36:2 with both orthotopic (yellow arrowheads) and ectopic RPE (cyan arrowheads). R: Retina, Ch: Choroid, d: Druse.

Autofluorescence images (Figs. 1*C*, 2, and 4) demonstrate that both ectopic and orthotopic RPE emit fluorescence at 572 nm when excited at 545 nm, whereas the neurosensory retina does not emit fluorescence under the same conditions. This property allows localization of ectopic RPE cells, which typically appear round, either isolated or in clumps.

**Fig. 3.**
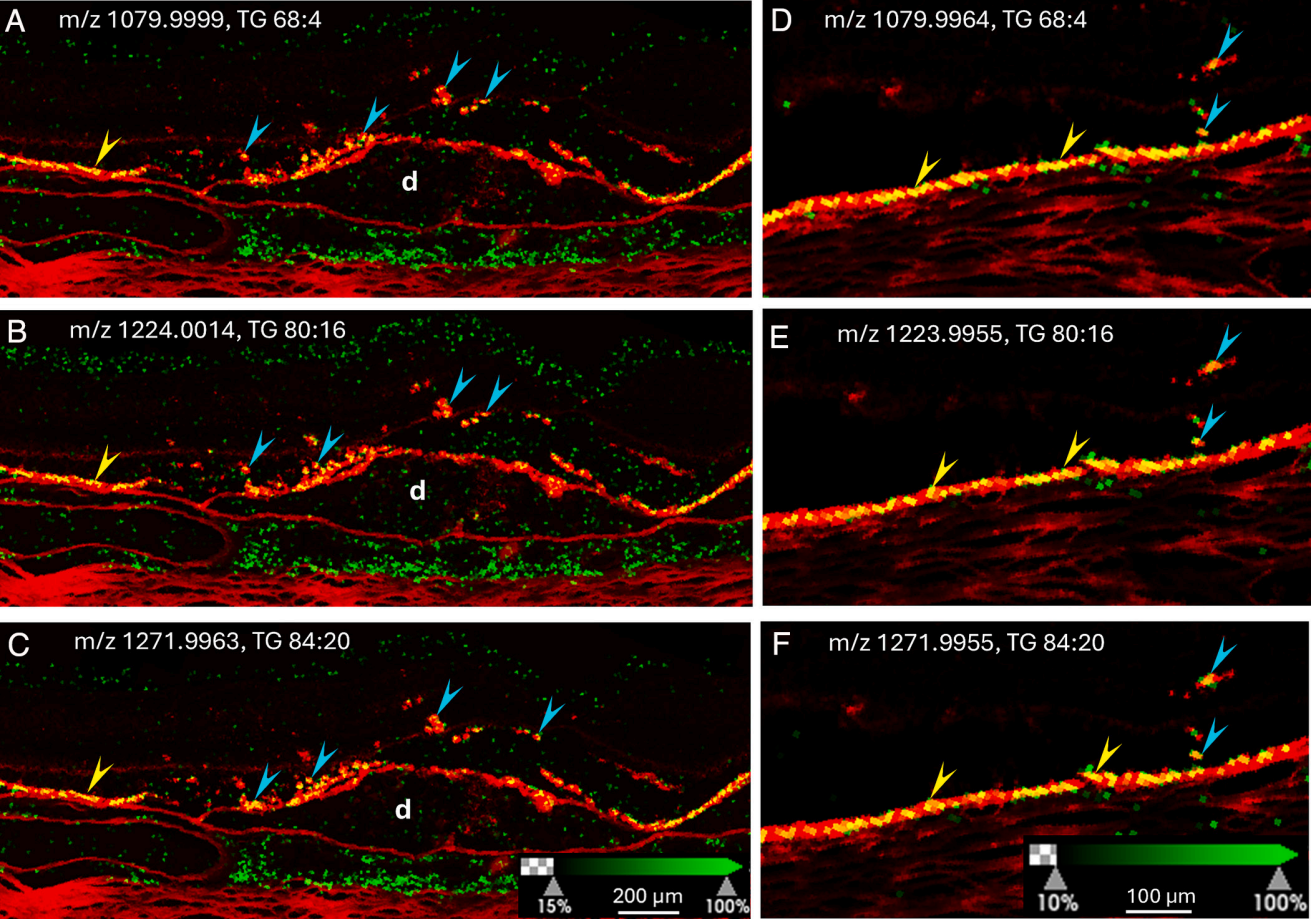
TGs are present in orthotopic and ectopic RPE cells. Overlays of autofluorescence signal (red) and IMS signal (green) in samples obtained from Donor 2 (*A*–*C*) and Donor 3 (*D*–*F*). Multiple TGs were detected (positive ion mode) in both orthotopic (yellow arrowheads) and ectopic RPE cells (cyan arrowheads). D: Druse.

Following coregistration of IMS and autofluorescence images, we searched for signals specific to orthotopic RPE cells, of which some were also detected in ectopic RPE cells. Results suggest that ectopic RPE cells retain certain molecular characteristics of native RPE. As shown in Fig. 2 (Donors 1 and 2), a signal at m/z 773.5339, identified as glycerophosphoglycerol (PG) 36:2, was detected in both orthotopic and ectopic RPE cells. Several triacylglycerols (TGs) with very-long-chain fatty acids were also found in both orthotopic and ectopic RPE cells. Representative signals are shown in Fig. 3 (Donors 2 and 3). Other TGs seen in both orthotopic and ectopic RPE cells include TG 66:2, TG 64:0, TG 72:8, TG 78:14, and TG 82:18. Overall, the presence of signals specific to orthotopic RPE cells in ectopic RPE supports their RPE origin. The detection of TGs with very-long-chain polyunsaturated fatty acids (VLC-PUFAs) in RPE is consistent with a prior report (35). It was previously suggested that RPE conserves and recycles long chain polyunsaturated fatty acids to the neural retina, where most VLC-PUFAs are found (36).

### Glycosphingolipids (GSLs) in Orthotopic and Ectopic RPE Cells.

**Fig. 4.**
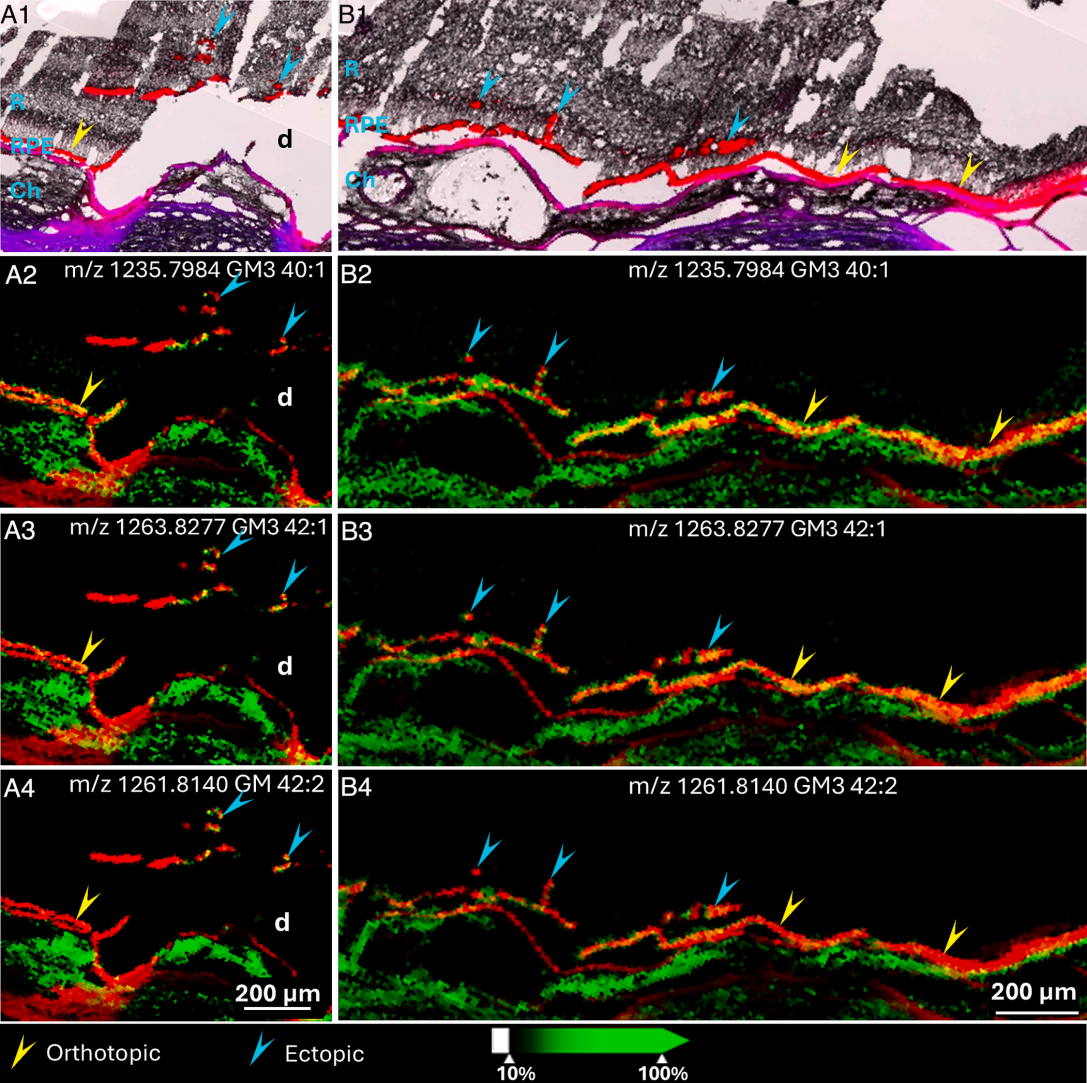
Detection of ganglioside species in choroid and RPE cells. Overlays of autofluorescence signal (red) and brightfield images (*A1* and *B1*) from Donor 1 show the presence of normal orthotopic RPE cells (yellow arrowheads) and ectopic RPE cells (cyan arrowheads). Bruch’s membrane is shown in purple under RPE (overlay DsRed and DAPI channels). Three ganglioside (GM3 40:1, GM3 42:1, and GM3 42:2) signals detected by negative ion mode IMS (green) are overlaid over autofluorescence signals (red) in two groups of ectopic RPE (*A1*–*A4* and *B1*–*B4*) and orthotopic RPE (yellow arrowheads). GM3 40:1 is abundant in orthotopic RPE cells and some ectopic RPE cells adjacent to orthotopic RPE layer (*B2*, cyan arrowheads) but barely detected in other ectopic RPE cells (*A2*, cyan arrowheads). GM3 42:1 is detected in both orthotopic and ectopic RPE cells (*A3* and *B3*) and GM3 42:2 signal is strong in some ectopic RPE cells migrating further into the neural retina (*A4*, cyan arrowheads) but decreased in orthotopic (yellow arrowheads) and minimally migrated RPE cells (*B4*, cyan arrowheads). R: Retina, Ch: Choroid, d: Druse.

Among signals detected in RPE cells, three were identified as GM3 gangliosides that localized to both orthotopic and ectopic RPE cells, as shown in Fig. 4 (Donor 1). However, the relative abundance of these gangliosides varied across different populations of ectopic RPE cells.

GM3 40:1 at m/z 1235.7984 was abundant in orthotopic RPE cells (Fig. 4, yellow arrowheads) and present in some ectopic RPE cells (Fig. 4*B2*, cyan arrowheads) but absent from others (Fig. 4*A2*, cyan arrowheads). Compared with GM3 40:1, the signal of GM3 42:1 at m/z 1263.8287 was higher in ectopic RPE cells and lower in orthotopic RPE (Fig. 4 *A3* and *B3*). GM3 42:2 at m/z 1261.8140 was barely detected in orthotopic RPE cells and was of low abundance in some ectopic RPE near the RPE layer but more prevalent in other ectopic cells (Fig. 4 *A4* and *B4*). Similarly, these three gangliosides were also detected in the retina from Donor 2 (*SI Appendix,* Fig. S1). In this case, all three GM3 signals were weak or undetectable in orthotopic RPE cells but were present in most ectopic RPE cells. Box and whisker plots for these gangliosides in samples from Donors 1 and 2 are shown in *SI Appendix*, Fig. S2. This quantitative analysis supports the heterogeneous distribution of these gangliosides in ectopic RPE of Donor 1 and consistently higher abundances in ectopic RPE in Donor 2. This heterogeneity may correspond to various stages of RPE transdifferentiation. Note that strong signals for these gangliosides were present in the choroid (Fig. 4 and *SI Appendix*, Fig. S1) and in drusen (*SI Appendix*, Fig. S1).

**Fig. 5.**
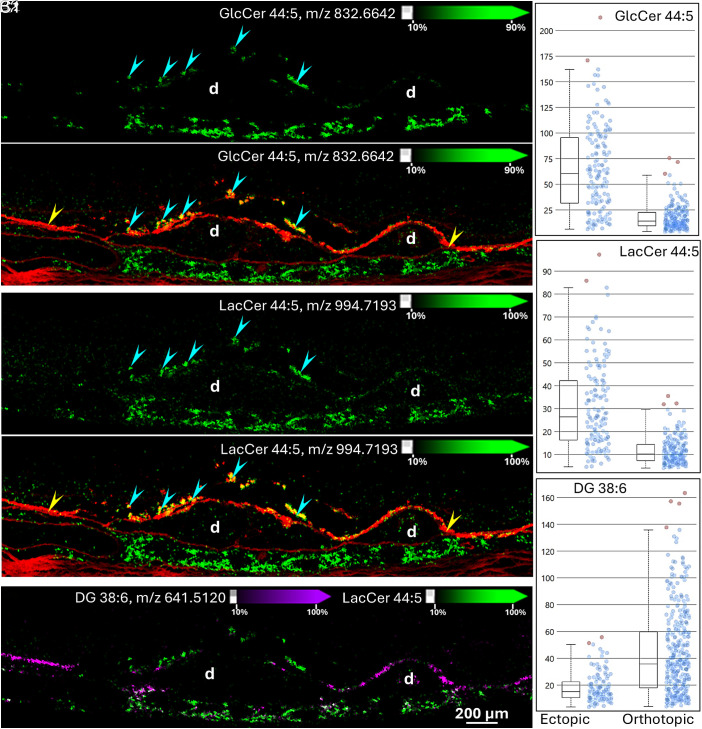
Detection of GlcCer and LacCer lipids in ectopic RPE cells. IMS signals acquired from Donor 2 in positive ion mode corresponding to m/z 832.6642, identified as GlcCer 44:5 (green, *A1, A2*) and m/z 994.7193, identified as LacCer 44:5 (green, *B1, B2*) were detected in ectopic RPE cells. IMS signal of GlcCer 44:5 is overlaid over red autofluorescence in panel *A2* showing colocalization with ectopic RPE cells (cyan arrowheads). Panel *B2* shows the overlaid LacCer 44:5 signal (green) with autofluorescence signal of ectopic RPE (cyan arrowhead). Panel *C1* shows the LacCer 44:5 signal (green) overlaid with m/z 641.5120, identified as DG 38:6 (purple), a signal that was present in orthotopic RPE (yellow arrowheads) but barely detected in ectopic RPE. Box and whisker plots present the intensities (*y*-axes) of GlcCer 44:5 (panel *A3*), LacCer 44:5 (panel *B3*), and DG 38:6 (panel *C2*) in each pixel of corresponding regions. Red dots represent the spots with intensities outside the intensity intervals (defined as 0 to 99%). By a Kruskal–Wallis test, all intensities differ significantly between ectopic and orthotopic RPE (*P* < 0.001). d: Drusen.

Besides these three gangliosides, we also detected a glucosylceramide (GlcCer 44:5) and lactosylceramide (LacCer 44:5) in ectopic RPE cells (Fig. 5, Donor 2). These GSLs were either undetectable or barely detectable in orthotopic RPEs. Overlays of LacCer 44:5 (m/z 994.7213) and diacylglycerol (DG) 38:6 (m/z 641.5137) are provided in Fig. 5*C1* DG 38:6 was detected in orthotopic RPE and at much lower intensity in ectopic RPE. LacCer 44:5 was not detected in regions with an attached and uniform layer of orthotopic RPE cells. The intensities of these lipids in orthotopic and ectopic RPE differed significantly, according to a Kruskal–Wallis test. As key precursors of GSL biosynthesis, the elevated levels of LacCer and GlcCer further indicate changes in GSL metabolism in ectopic RPE.

### Loss of Phosphatidylinositol (PI) Species in Ectopic RPE Cells.

**Fig. 6.**
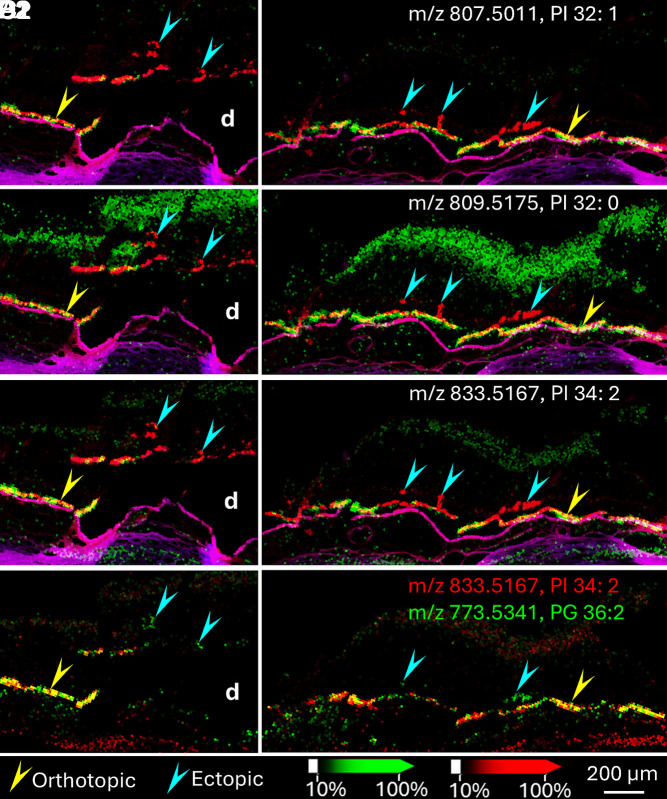
PI species in RPE cells. PI lipid signals detected in RPE cells (green) by IMS from Donor 1 in negative ion mode are overlaid over autofluorescence signals (red) (*A1* and *A2*, PI 32:1; *B1* and *B2*, PI 32:0; *C1* and *C2*, PI 34:2). Bruch’s membrane is shown in purple (overlay autofluorescence of DsRed and DAPI channels). These PI lipids were detected in orthotopic RPE (yellow arrowheads) but not in ectopic RPE cells (cyan arrowheads). *D1* and *D2* shows an overlay of PI 34:2 (red) with PG 36:2 (green, m/z 773.5341) (a signal in both orthotopic and ectopic RPE) indicating no signal for PI 34:2 in ectopic RPE. d: Druse.

**Fig. 7.**
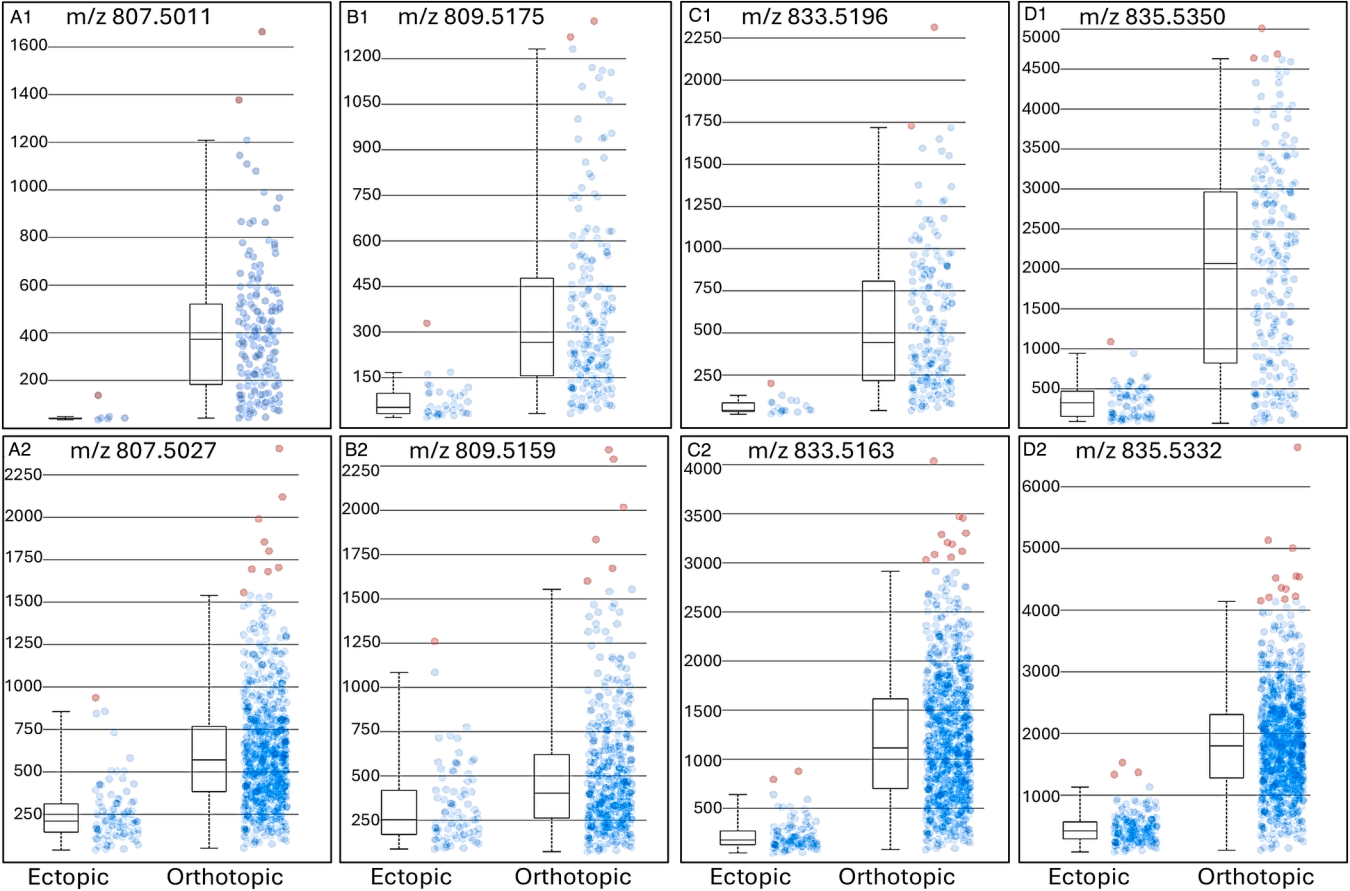
Box and whisker plots of intensities of four PI lipids in orthotopic and ectopic RPE. Intensity box and whisker plots for four PI lipids—PI 32:1 (m/z 807.5011), PI 32:0 (m/z 809.5175), PI 34:2 (m/z 833.5167), and PI 34:1 (m/z 835.5340) are shown for Donor 1 (*A1*–*D1*) and Donor 2 (*A2*–*D2*). Mass interval setting is 5 ppm. The y-axes display the intensities of the selected m/z in each spot within selected regions. Red dots represent the spots with intensities outside the intensity intervals (defined as 0 to 99%). These results support the conclusion that ectopic RPE cells exhibit a loss of RPE-abundant PI lipids. By a Kruskal–Wallis test, all intensities differ significantly between ectopic and orthotopic RPE (*P* < 0.001).

Previously, we found highly abundant PI lipids in RPE of healthy central retina (34). Consistently, we detected three PI lipids (PI 32:1, PI 32:0, and PI 34:2) that are abundant in orthotopic RPE and undetectable in ectopic RPE (Fig. 6, Donor 1). Consistent with our previous report (34), PI lipids differing by a single double bond showed markedly different spatial distributions. For example, in the neurosensory retina, PI 32:1 was barely detected and PI 32:0 showed strong signal. Similarly, PI 34:1 is also highly abundant in the retina (*SI Appendix*, Fig. S3) and orthotopic RPE but is undetectable in ectopic RPE. Other PI lipids are either barely detected in RPE (PI 38:6, *SI Appendix*, Fig. S3) or are highly abundant in retina, which impedes finding a distinction between orthotopic and ectopic RPE. The disappearance of PI 34:2 signal in ectopic RPE cells is further illustrated by overlaying signals of PI 34:2 with PG 36:2 (Fig. 6 *D1* and *D2*). PG 36:2 was detected in both orthotopic and ectopic RPE cells, but PI 34:2 was not detected in ectopic RPE cells. This finding was replicated in a second sample (*SI Appendix*, Fig. S4, Donor 2). Box and whisker plots of PI lipid intensities (Fig. 7, Donors 1 and 2) demonstrate PI lipids in ectopic RPE have significantly lower signal than those in the orthotopic RPE, confirming the results shown in Fig. 6.

## Discussion

This study aimed to identify molecular signals that either unify or differentiate orthotopic and ectopic RPE cells, and elucidate cellular sources of HRF and mechanisms driving RPE migration in AMD. Our multimodal imaging study has confirmed a portion of HRF represents cells of RPE origin. For example, ectopic RPE cells appear pigmented in brightfield images and share similar autofluorescence properties to normal RPE cells. Additionally, our IMS analysis detected lipid signals common to both orthotopic and ectopic RPE cells and not in surrounding cells. These results support previous reports of RPE cell migration into the neurosensory retina (7, 8, 20). As evidence increasingly supports RPE transdifferentiation and migration (30, 37), identifying factors that trigger RPE migration is therefore crucial. Advances in this area will contribute to developing strategies to maintain RPE function and monolayer integrity, which are essential for the health of the retina, choroid, and overall ocular homeostasis. Data can also inform the development of new model systems for retinopathy research and inspire new treatment approaches.

Our IMS findings suggest that lipid signals vary across different populations of ectopic RPE cells. These results are consistent with previous reports suggesting that RPE degeneration and migration are associated with heterogeneous molecular changes, potentially reflecting a staged progression (25, 38). IMS with near single-cell resolution has the potential to capture subpopulation-specific signals that correspond to such stages. However, the current study is based on a limited number of samples which precludes correlation analysis. Future studies involving IMS analysis of a larger sample size, combined with autofluorescence and histology imaging to obtain the height of pigmented cell migration into the neurosensory retina, could help identify specific molecular pathways driving RPE migration. Our data, while identifying glycolipids as mediators, do not exclude a role for hypoxia in inducing RPE migration, as HRF are prominently seen atop large drusen, far from choriocapillaris microvasculature (39, 40).

One type of lipid detected in our study that is differentially populated in ectopic RPE and orthotopic RPE cells is GSLs. Our IMS results suggest that the levels of three gangliosides vary across ectopic RPE at different stages of migration, supporting a potential role of these GSLs in driving RPE migration. Substantial evidence now supports the involvement of EMT (8, 27, 41, 42), during RPE migration. EMT is a transdifferentiation process characterized by multiple cellular events, including reduced cell–cell adhesion, increased expression of mesenchymal markers, and decreased expression of epithelial factors (43). Previously, GSLs have been shown to undergo dynamic expression changes during EMT and may play an important role in regulating intercellular adhesion (43, 44). Transforming growth factor beta (TGF-β1), a strong EMT promotor, has been reported to increase the intracellular levels of LacCer, and a-series gangliosides (GM3, GM2, and GM1a) in NM18 cells (44). Similar to TGFβ, transcription factor Zeb1 influences cell adhesion and promotes expression of a-series GSLs (44). In another study, it was reported that ganglioside GM3 potentially interacts with TGF-β receptors in HLE B-3 cells and participates in TGF-β1-induced EMT (45).

Our results show the presence of GM3 gangliosides (40:1, 42:1, and 42:2) in RPE cells. This result comports with previous findings that GM3 gangliosides, especially ceramides with 40 and 42 carbons, are present in RPE cells (46). Since we did not simultaneously analyze IMS signals from healthy retina tissue, we cannot determine whether the level of GM3 gangliosides is elevated in AMD donors. Although GM3 40:1 and 42:1 were detected in a continuous RPE layer that was still attached to Bruch’s membrane, we do not know whether lipid metabolism has started to change in those cells. Therefore, accurately quantifying the levels of these gangliosides in retina from normal and AMD donors could provide further valuable insights toward understanding whether these gangliosides are involved in driving RPE migration.

Our results also revealed LacCer 44:5 and GlcCer 44:5 as two lipids that are detected only in ectopic RPE cells. LacCer, a key signaling lipid, activates cytosolic phospholipase A2, which cleaves arachidonic acid to generate prostaglandins and induce inflammation (47, 48). Additionally, LacCer has been shown to induce reactive oxygen species (ROS) production by activating NADPH oxidase (49). Both inflammation and ROS have been linked with EMT and RPE migration (13, 43, 49). Moreover, as common biogenic precursors of GSLs such as gangliosides, globosides, and sulfatides, the accumulation of LacCer and GlcCer in ectopic RPE cells further suggests that GSL metabolism is associated with RPE migration.

Another important finding revealed by IMS is that ectopic RPE cells lacked some PI lipids that are abundant in orthotopic RPE cells. PI lipids constitute 6.5% of total phospholipid in RPE cells, which is higher than their abundance in photoreceptors OS (50). Besides being key membrane components, PI lipids serve as precursors for several important signaling molecules such as phosphoinositides (PIPs), DG, and 1,4,5-inositol triphosphate (51). These molecules regulate a broad range of processes, including cell signaling, proliferation and differentiation, phagocytosis, and phototransduction 52, 53. Moreover, PIPs also play important roles during EMT. For instance, PI(4, 5)P2 accumulates in lipid rafts and modulates actin and the actin-binding proteins α-actinin, cofilin, and villin to influence cell surface protrusive motility (52, 54). PI(4, 5)P2 has also been proposed to dynamically regulate focal adhesion assembly via protein interactions (53). Additionally, PI(4, 5)P2 is known to regulate endothelial growth factor (EGF)-stimulated directional cell migration, adhesion dynamics, and cytoskeleton rearrangement (55).

Considering the potential role of PIPs in EMT, the nondetectability of PI lipids in ectopic RPE cells could be attributed to phosphorylation of PI lipids to PIPs. Recently, phosphoinositide 3-kinase/Akt signaling pathway has been reported to be up-regulated in human RPE cells following treatment with TGFβ2, a growth factor that promotes EMT (56). However, we did not detect PIPs signals corresponding to those PI lipids that are absent in the ectopic RPE. Note that PIPs are generally low in abundance and have lower detectability than Pl lipids, with their detectability decreasing significantly in matrix-assisted laser desorption/ionization (MALDI) analysis as the number of phosphate groups increases (57). Therefore, we cannot determine presence or absence of PIPs based on lack of IMS signals. It is essential to develop more sensitive methods to test whether the formation of PIPs is upregulated in ectopic RPE.

In conclusion, by leveraging the high spatial resolution and untargeted approach of IMS, we identified lipid signal differences between ectopic RPE and adjacent orthotopic RPE. Our results revealed altered metabolism of GSLs and PI lipids, with all observed changes potentially linked to inflammation and EMT. At the level of spatial resolution used herein, characterizing molecular signatures of RPE with different migration heights (58) into the neurosensory retina becomes feasible. However, increased spatial resolution comes at the expense of sensitivity. To preserve tissue integrity, typical washing steps to remove salts and to improve sensitivity before MALDI analysis were omitted. Additional limitations include the inability to assign sn-chain identification or double bond positions in detected lipids and the number of samples analyzed. Further, because a common MALDI matrix for lipid analysis was used, certain lipids, such as cholesterol and cholesterol esters known from drusen, may have been poorly detected. However, due to rapid advancements in sample preparation, instrument sensitivity, and MALDI matrices (59), integration of MALDI IMS with OCT and other imaging tools will continue to provide valuable insights into RPE migration and aid in identifying potential targets to halt or retard the causative cellular processes.

## Methods

### Collection and Characterization of Human Donor Eyes.

Whole eyes were obtained from deceased human donors by Advancing Sight Network (Birmingham, AL) as part of ongoing studies on AMD that were approved by institutional review at University of Alabama at Birmingham (protocol # N170213002) where tissues were collected. The eye bank was requested to provide whole eyes from donors >80 y of age that could be received in the laboratory <6 h after death. In total, five donor eyes were used in this study (Table 1). Whole globes were opened anteriorly, immersed in 4% phosphate-buffer paraformaldehyde overnight, and stored long-term in 1% paraformaldehyde at 4 °C. All eyes were examined with multimodal ex vivo fundus imaging including color and OCT (Spectralis, Heidelberg Engineering, Heidelberg Germany), as described (30° macula cube, 30 μm spacing, averaging = 25) (60). Samples used for analysis had HRF within the 6-mm-diameter central retina on ex vivo OCT, in the setting of drusen and in some cases, subretinal drusenoid deposits (Table 1). Ex vivo OCT images for samples used in this study can be found in the *SI Appendix*, Fig. S5.

### Tissue Preparation.

Retina tissues were prepared as previously reported (34). Dissected tissue containing central retina with the fovea in it was embedded in 2.25% carboxymethylcellulose before sectioning at 12 to 14 µm thickness and mounting onto indium tin oxide coated microscope slides (Delta Technologies, Loveland, CO, USA). Slides were stored at −80 °C, then brought to room temperature in a vacuum desiccator before analysis. Sections near those used for MALDI-IMS were collected on glass slides and stained with PASH to identify drusen and basal laminar deposit (PASH, Poly Scientific R&D Corp., Bay Shore, NY, USA; #K047 kit). Slides were dehydrated through 85%, 95%, 100% ethanol, and 100% xylene (Fisher, # X3S-4) for 5 min, twice at each concentration. All slides were cover-slipped with permanent medium (Permount, EMS, # 17986-01) and air-dried in a hood overnight. Stained sections were scanned with a 40× objective.

### Microscopy.

Three channel epifluorescence images and brightfield images were acquired before and after MALDI IMS analysis using a fluorescence slide scanner equipped with a light-emitting diode light source (AxioScan.Z1, Colibri7, Carl Zeiss Microscopy GmbH, Oberkochen, Germany). Three channels (excitation, emission) include DAPI (353 nm, 465 nm), EGFP (488 nm, 509 nm), and DsRed (545 nm, 572 nm). Post IMS autofluorescence images were also collected to visualize the grid pattern of MALDI laser burns (34) and thus register the images.

### MALDI Matrix Application and MALDI IMS Analysis.

Matrix material was applied to tissue section using an in-house developed sublimation device as described in the supplemental materials. Matrices included 2,5-dihydroxyacetophenone (DHA) (positive mode), or 1,5-diaminonaphalene (DAN) (negative mode) (Sigma-Aldrich, St. Louis, MO), or 4-(dimethylamino) cinnamic acid (DMACA) (both positive and negative mode). MALDI IMS data were acquired with a 5 to 10 μm pixel size using a Trapped Ion Mobility Spectroscopy–Time of Flight (timsTOF) Flex MALDI imaging platform with TIMS deactivated (Bruker Daltonics, Bremen, Germany). The mass spectrometer was calibrated with red phosphorus prior to data acquisition using a 120,000 FWHM (at m/z 400) mass resolving power. Data were acquired in either positive or negative ionization mode within a mass range of m/z 400 to 1500 with 100 laser shots per pixel.

### MALDI IMS Data Processing.

timsTOF data were internally recalibrated using DataAnalysis software (Bruker Daltonics, Bremen, Germany) and were loaded into SCiLS lab MVS (version 2024b Pro; Bruker Daltonics, Bremen, Germany). Features were extracted using **T**ime-aligned-**R**egion-complete-**eX**traction feature finding with 1% relative intensity available in SCiLS Lab. Total ion current was used for normalization. Coregistration was performed in SCiLS Lab. A detailed description can be found in the supplemental materials. Signals colocalizing with each manually annotated region were determined based on the Pearson correlation in SCiLS. Ion images identified by SCiLS were inspected manually and were overlaid over autofluorescence or bright-field images to show colocalization. For quantification, regions corresponding to orthotopic and ectopic RPE were manually annotated in SCiLs Lab based on the DsRed channel of the autofluorescence signal as coregistered with IMS. Ion intensities in each pixel were used for comparing lipid abundances across different ROIs. Intensities of lipid signals in ectopic and orthotopic RPE were compared within the SCiLS lab MVS environment using the Kruskal–Wallis test for nonnormally distributed data; *P* < 0.001 was considered significant.

### Lipid Identification.

Signals of interest from timsTOF analysis were exported from SCiLS and searched against the LIPIDMAPS Database (http://www.lipidmaps.org) and annotated based on accurate mass (within 5 ppm). Generally, [MH]^+^ ions were considered in the positive mode, and [MH]^−^ ions were considered in the negative mode. [M + H-H_2_O]+ and [M + Na]+ ions were also evaluated in positive mode to account for lipids that typically lose water or form sodium adducts in positive mode.

## Supplementary Material

Appendix 01 (PDF)

## Data Availability

All study data are included in the article, the *SI Appendix* or are available at: https://doi.org/10.7910/DVN/HIVYWA (61).
