# Supplementary material for: Glycolipids implicated as mediators of clinically visible retinal pigment epithelial migration in age-related macular degeneration
Source: Proc Natl Acad Sci U S A. 2025 Jul 14;122(29):e2503191122. doi: 10.1073/pnas.2503191122 (PMC12305059; doi:10.1073/pnas.2503191122)
Supplement: Supplementary file 1 — Appendix 01 (PDF) [file pnas.2503191122.sapp.pdf]

PNAS

Supporting Information for

Glycolipids implicated as mediators of clinically visible retinal pigment epithelial migration in age-related macular degeneration.

Zhen Wang[a,1], David M.G. Anderson[a,1], Jeffrey D. Messinger[b], Christine A. Curcio[b], Kevin L. Schey[a,2]

[a] Department of Biochemistry and Mass Spectrometry Research Center, Vanderbilt University, Nashville, TN, 37532
[b] Department of Ophthalmology and Visual Sciences, University of Alabama at Birmingham Heersink School of Medicine, Birmingham, AL, 35233

[1] The authors contributed equally to this work
[2] To whom correspondence may be addressed.
Email: k.schey@vanderbilt.edu

This PDF file includes:

Figures S1 to S5

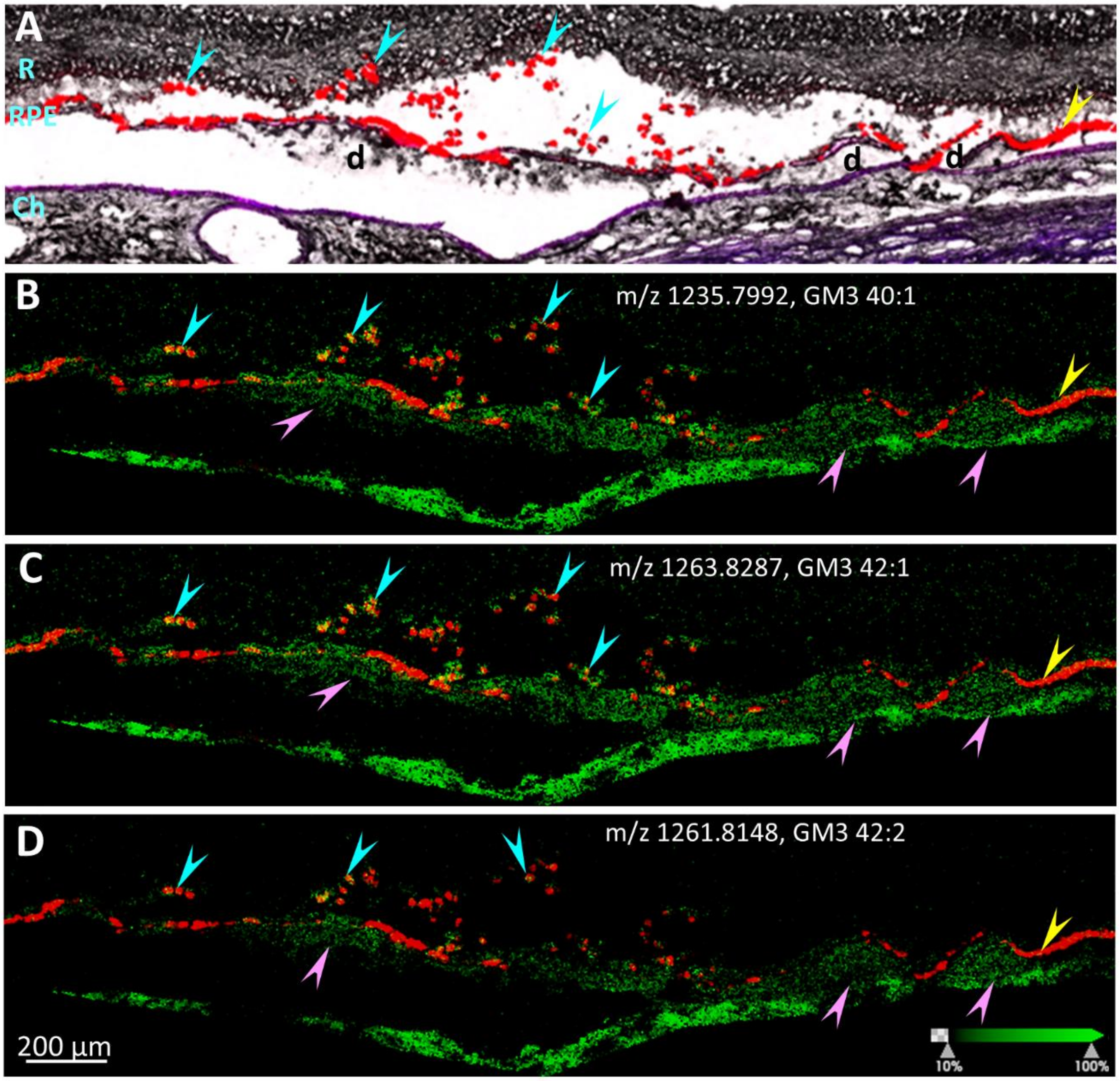

Fig. S1. Detection of ganglioside species in ectopic RPE cells in Donor 2.

An overlay of the brightfield image with autofluorescence image (red) from Donor 2 demonstrates the presence of ectopic RPE cells and drusen (d). Ectopic RPE cells migrate to the subretinal space with some entering the neurosensory retina (cyan arrowheads). Orthotopic RPE (yellow arrowhead at the right) is continuous and even in height. (B, C, D) Three ganglioside signals detected by IMS (green) (negative ion mode) are overlayed over the autofluorescence signals (red). GM3 40:1 (B) and GM3 42:1 (C) are weakly detected in orthotopic RPE cells (yellow arrowhead) but the signals are detected in ectopic RPE cells (cyan arrowheads). (D) GM3 42:2 signal was detected in some ectopic RPE cells and is not detected in orthotopic RPE (yellow arrowhead). All three gangliosides are present in drusen (pink arrowheads) and choroid. R: Retina; Ch: Choroid.

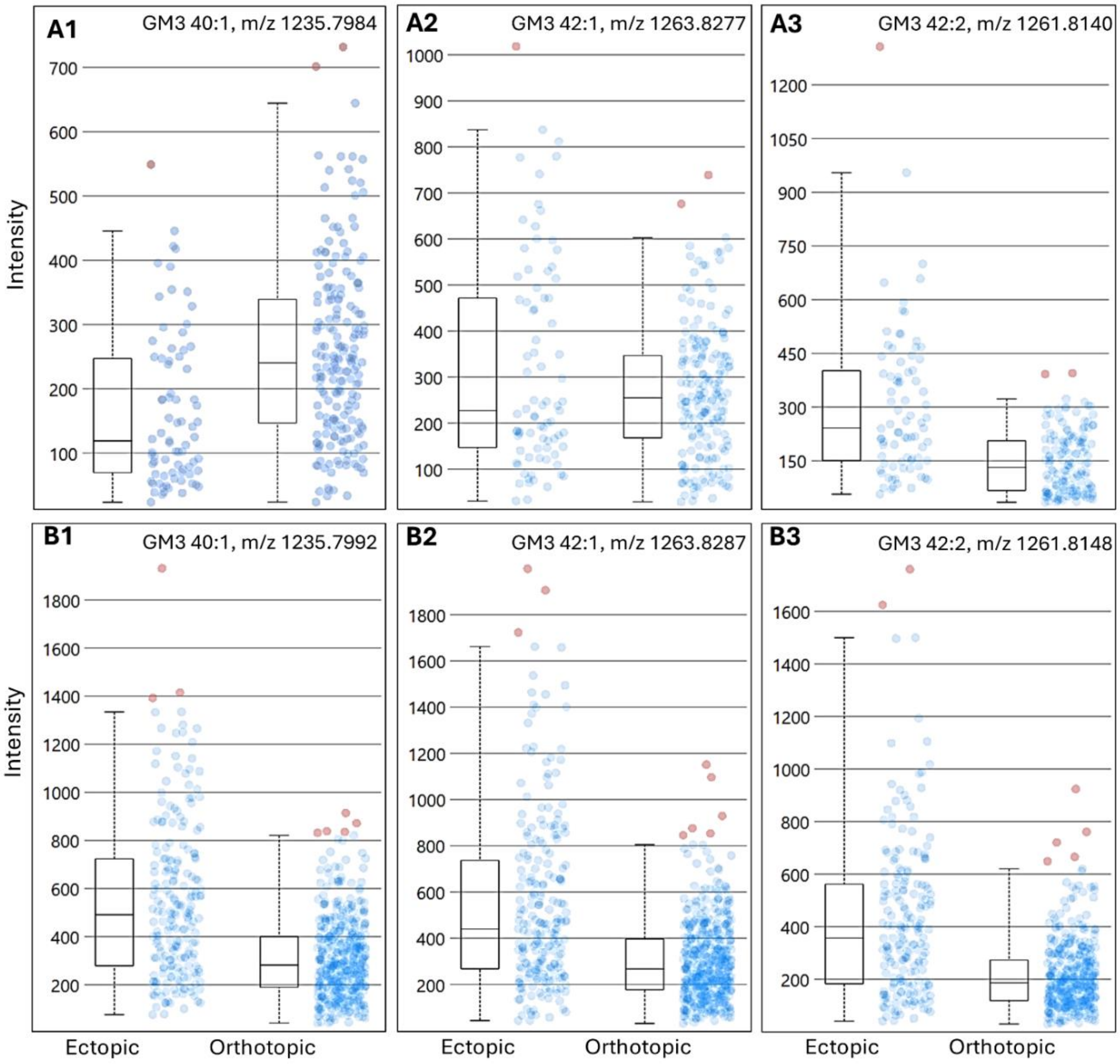

Fig. S2. Box and whisker plots for three gangliosides in Donor 1 and Donor 2.

Intensity box and whisker plots for three gangliosides GM3 40:1 (A1, B1), GM3 42:1 (A2, B2) and GM3 42:2 (A3, B3) are shown for Donor 1 (A1-A3) and Donor 2 (B1-B3). Mass interval setting is 5 ppm. The y-axes display the intensities of the selected m/z in each spot within selected regions. Red dots represent the spots with intensities outside the intensity intervals (defined as 0-99%). By a Kruskal-Wallis test performed using SCiLS software for IMS analysis, GM3 42:2 is significantly higher in ectopic RPE in Donor 1, but intensities of GM3 40:1 and GM3 42:1 are not significantly different between ectopic and orthotopic RPE, however, Intensities of all three gangliosides are significantly higher in ectopic than orthotopic RPE in Donor 2 ($p < 0.001$).

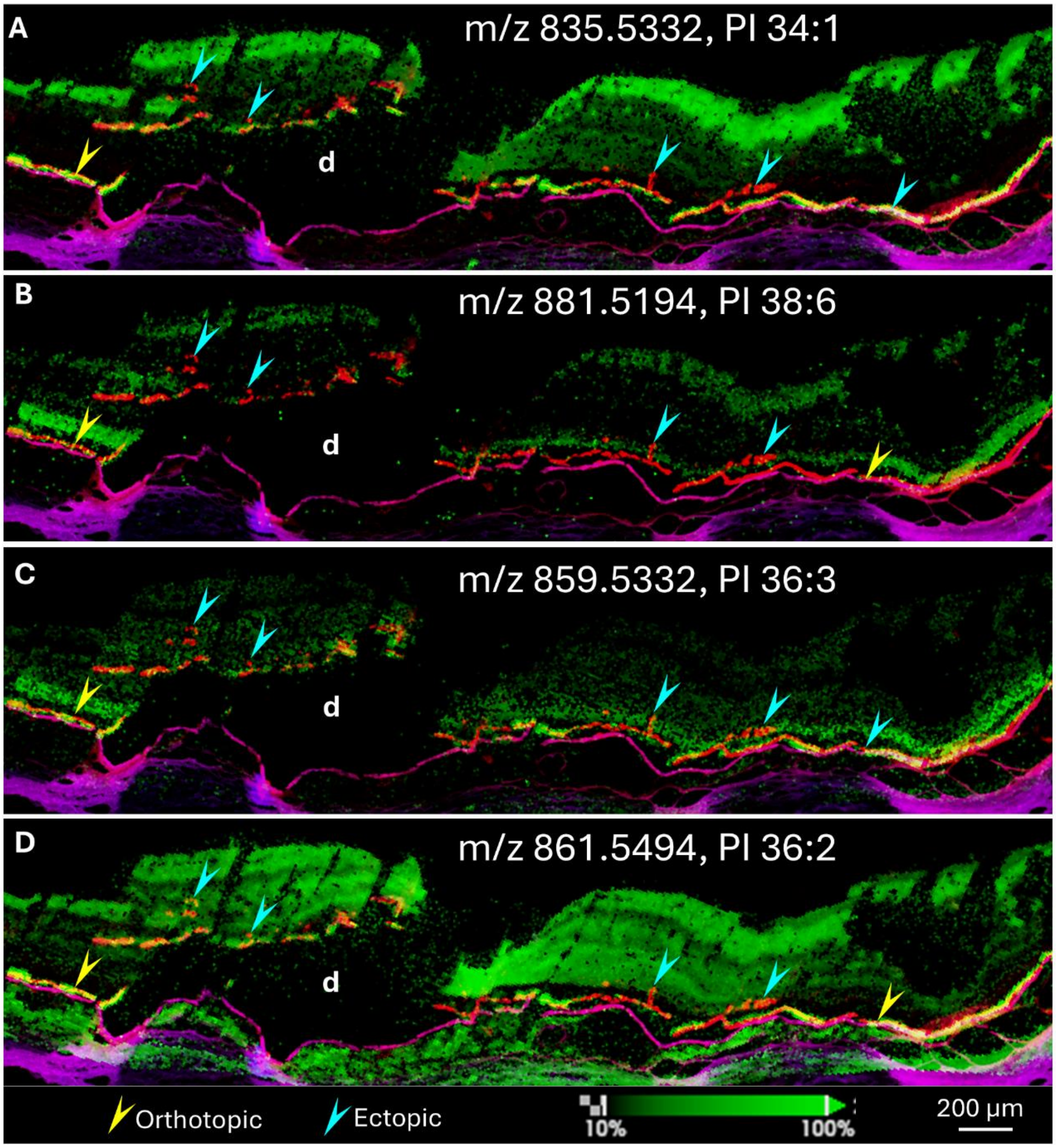

Fig. S3. Localization of other phosphatidylinositol (PI) lipids in Donor 1.

(A,B,C,D) Four PI lipid signals detected by IMS (green) of Donor 1 in negative ion mode are overlayed over autofluorescence signals (red). PI lipids differing by a single double bond exhibit strikingly different distribution in the retina. The absence of PI lipids signals in ectopic RPE is evident for PI 34:1, PI 38:6. (D) The strong signals of PI 36:3 and PI 36:2 in the retina makes it difficult to see whether these lipids are present in ectopic RPE. Purple color shows the overlay of DsRed and DAPI signals in Bruch’s membrane and choroid. Drusen: d.

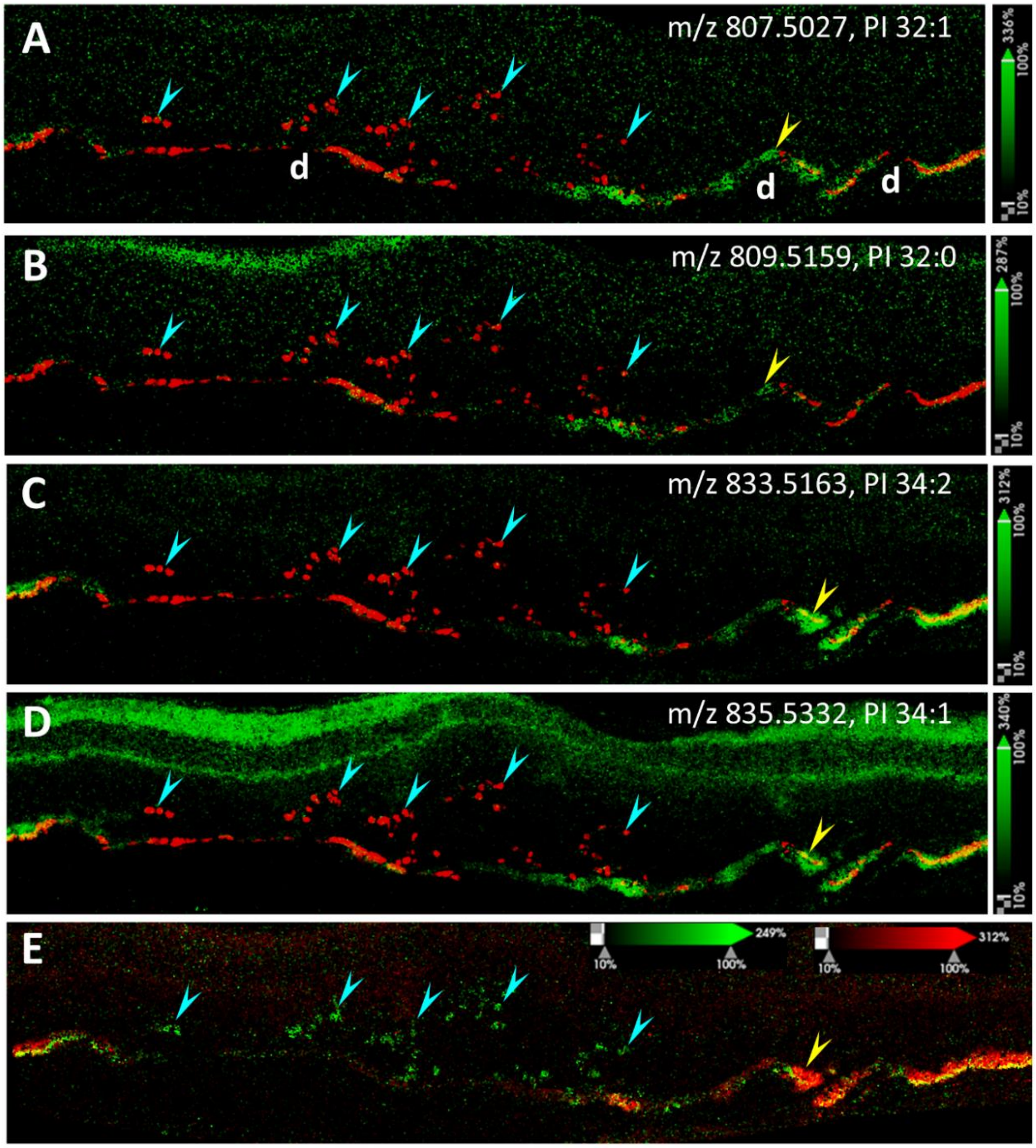

Fig. S4. Phosphatidylinositol species in RPE cells in Donor 2.
(A,B,C,D) Four PI lipid signals detected in RPE cells by IMS (green) of Donor 2 in negative ion mode are overlayed over autofluorescence signals (red) (A, PI 32:1; B, PI 32:0; C, PI 34:2, and D, PI 34:1). These PI lipids were not detected in ectopic RPE cells (pink arrowheads). Panel E shows an overlay of PI 34:2 (red) with PG 36:2 (green, m/z 773.5339) (a signal in both orthotopic and ectopic RPE) indicating no signal of PI34:2 in ectopic RPE. Drusen: d.

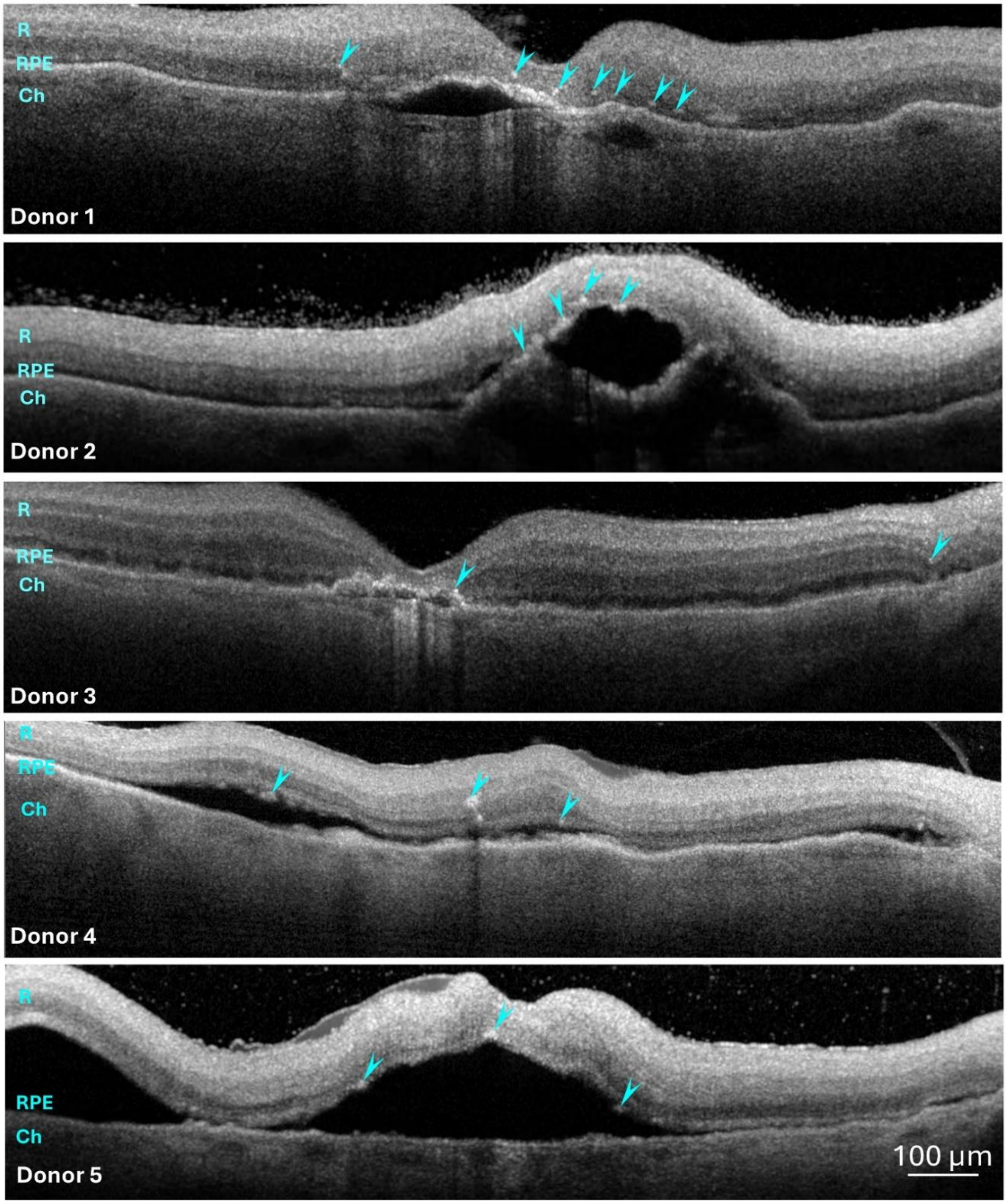

Fig. S5. Ex vivo OCT images for samples used in this study.

Images were created by removing cornea and lens from a preserved donor eye and placing it in an eye holder that "looks" into an OCT device. The retina is at the top of each scan, and the choroidal vasculature is at the bottom. The optic nerve is off the right edge of the scan. Hyperreflective foci are indicated by cyan arrowheads. Cryosections from these five right eyes were used for analysis by imaging mass spectrometry and multimodal microscopy. R: Retina; Ch: Choroid.
